# Increased availability of NADH in metabolically engineered baker’s yeast improves transaminase-oxidoreductase coupled asymmetric whole-cell bioconversion

**DOI:** 10.1186/s12934-016-0430-x

**Published:** 2016-02-15

**Authors:** Jan Dines Knudsen, Cecilia Hägglöf, Nora Weber, Magnus Carlquist

**Affiliations:** Division of Applied Microbiology, Department of Chemistry, Faculty of Engineering, Lund University, PO Box 124, 221 00 Lund, Sweden; The Department of Biotechnology and Biosciences, University of Milano-Bicocca, P.zza della Scienza 4, 20126 Milano (MI), Italy; Evolva, Duggingerstrasse 23, 4153 Reinach, Switzerland

**Keywords:** Whole-cell biocatalysis, Co-factor regeneration, Chiral amines, Chiral alcohols, Glycerol-3-phosphate dehydrogenase, Kinetic resolution, (*R*)-1-phenylethylamine, (*S*)-1-phenylethanol

## Abstract

**Background:**

*Saccharomyces cerevisiae* can be engineered to perform a multitude of different chemical reactions that are not programmed in its original genetic code. It has a large potential to function as whole-cell biocatalyst for one-pot multistep synthesis of various organic molecules, and it may thus serve as a powerful alternative or complement to traditional organic synthetic routes for new chemical entities (NCEs). However, although the selectivity in many cases is high, the catalytic activity is often low which results in low space-time-yields. In the case for NADH-dependent heterologous reductive reactions, a possible constraint is the availability of cytosolic NADH, which may be limited due to competition with native oxidative enzymes that act to maintain redox homeostasis. In this study, the effect of increasing the availability of cytosolic NADH on the catalytic activity of engineered yeast for transamination-reduction coupled asymmetric one-pot conversion was investigated.

**Results:**

A series of active whole-cell biocatalysts were constructed by over-expressing the (*S*)-selective ω-transaminase (*VAMT*) from *Capsicum chinense* together with the NADH-dependent (*S*)-selective alcohol dehydrogenase (*SADH*) originating from *Rhodococcus erythropolis* in strains with or without deletion of glycerol-3-phosphate dehydrogenases 1 and 2 (*GPD1* and *GPD2*). The yeast strains were evaluated as catalysts for simultaneous: (a) kinetic resolution of the *racemic* mixture to (*R*)-1-phenylethylamine, and (b) reduction of the produced acetophenone to (*S*)-1-phenylethanol. For the *gpd1*Δ*gpd2*Δ strain, cell metabolism was effectively used for the supply of both amine acceptors and the co-factor pyridoxal-5′-phosphate (PLP) for the ω-transaminase, as well as for regenerating NADH for the reduction. In contrast, there was nearly no formation of (*S*)-1-phenylethanol when using the control strain with intact *GPD*s and over-expressing the *VAMT*-*SADH* coupling. It was found that a *gpd1*Δ*gpd2*Δ strain over-expressing *SADH* had a 3-fold higher reduction rate and a 3-fold lower glucose requirement than the strain with intact *GPD*s over-expressing *SADH*.

**Conclusions:**

Overall the results demonstrate that the deletion of the *GPD1* and *GPD2* genes significantly increases activity of the whole-cell biocatalyst, and at the same time reduces the co-substrate demand in a process configuration where only yeast and sugar is added to drive the reactions, i.e. without addition of external co-factors or prosthetic groups.

**Electronic supplementary material:**

The online version of this article (doi:10.1186/s12934-016-0430-x) contains supplementary material, which is available to authorized users.

## Background

Chiral amines and chiral alcohols are important functional moieties in many bioactive compounds and they are essential for completing total synthesis of various drugs [1]. There is thus a need for economically and environmentally sustainable methods for their preparation. Chiral amines and alcohols can be generated enzymatically from ketones by the action of ω-transaminases (ω-TAs) [2, 3] and ketone reductases (KREDs) [4], respectively. ω-TAs require the prosthetic group pyridoxal-5′-phosphate (PLP) for functionality and also a sufficient supply of amine acceptor or amine donor molecules depending on the mode of operation, i.e. kinetic resolution of *racemic* amines or direct amination of ketones. In most described biocatalytic transaminations, the required enzymes, PLP and amine acceptors/donors have been directly added to the reaction mixture [5]. Similarly, asymmetric reduction of ketones to chiral alcohols by KREDs require addition of the cofactor NADH or NADPH and a system for hydride recycling, e.g. by the action of formate dehydrogenase or glucose dehydrogenase.

Coupled transamination and reduction reactions can be used for conversion of alcohols to amines or for the reverse reaction, i.e. amines to alcohols, and are useful tools in the organic synthetic toolbox for functional modification and synthesis of new chemical entities (NCEs). The coupled two-step reactions can be catalysed in one-pot by living microbial cells that co-express the required enzymes and use cell metabolism for (re-)generation of co-factors, prosthetic groups, and essential co-substrates. Other strongholds of using microbial cells as catalysts include high selectivity, renewable origin and a simplified upstream processing, as well as operation under relatively mild and environmentally benign conditions [6]. In line with this, one-pot conversion of 1,10-decanediol to 1,10-diaminedecane was recently achieved in engineered *Escherichia coli* strains co-expressing alcohol dehydrogenase, transaminase, and alanine dehydrogenase [7]. Baker’s yeast *Saccharomyces cerevisiae* was previously engineered for kinetic resolution of *racemic* 1-phenylethylamine to (*R*)-1-phenylethylamine and simultaneous reduction of the formed acetophenone to (*R*)-1-phenylethanol [8]. The reactions were catalysed in vivo by a recombinant ω-TA from *Capsicum chinense* co-expressed with a recombinant NADPH-dependent alcohol dehydrogenase from *Lactobacillus kefir*. Both PLP and amine acceptors (for example pyruvate) for the ω-TA [9], as well as NADPH for the oxidoreductase [8] was previously shown to be possible to provide from cell metabolism. However, the engineered yeast biocatalysts suffered from low specific activity which leads to high loadings of yeast and sugar in order to reach high conversions.

A possible limiting factor for KRED-based reaction cascades is the availability of intracellular NAD(P)H [10, 11]. To increase the availability of NADPH, a number of in vivo systems in which the cellular carbon metabolism is used to fuel the oxidation-reduction cycle have been developed [12, 13]. For example, metabolically engineered *S. cerevisiae* with increased flux through the pentose-phosphate pathway resulting in an increased availability of NADPH for recombinant oxidoreductases have been described [14]. The NADPH platform was successfully used with different NADPH-dependent KREDs for preparative-scale production of chiral alcohols via whole-cell bioconversion of prochiral ketones [10, 15] or from the *racemic* mixtures [11, 16]. Other recent examples of engineered whole-cell systems in which the native cell metabolism was re-wired for an increased availability of NADPH is the cyclised pentose-phosphate pathway system in *Corynebacterium glutamicum* [17] and the elevated activity of transhydrogenase and NAD^+^ kinase in *E. coli* [18].

With regards to NADH in baker’s yeast, the NADH formed in the glycolysis during fermentative or respire-fermentative mode of metabolism is oxidized by alcohol dehydrogenases (ADHs) that catalyse the reduction of acetaldehyde to ethanol. Under oxygen-limited conditions, the additional cytosolic NADH formed from anabolic reactions such as during amino acid biosynthesis is oxidized by glycerol-3-phosphate dehydrogenase (GPD) isoenzymes 1 and 2, which catalyse the reduction of dihydroxyacetone phosphate (DHAP) to glycerol-3-phosphate [19, 20]. Thus, to fully exploit the cellular capacity to regenerate NADH required for whole-cell biocatalytic reactions, there may be a need to deactivate native NADH oxidating enzymes. It has previously been demonstrated that the limited capacity to oxidize NADH in *gpd1*Δ*gpd2*Δ strain can be exploited to achieve higher formation of xylitol [21, 22] and butanol [23].

In this study, the suitability of *gpd1*Δ*gpd2*Δ yeast to function as platform host for whole-cell biocatalytic transaminase-reductase coupled reactions was evaluated. For this purpose, the use of *gpd1*Δ*gpd2*Δ yeast for simultaneous preparation of chiral (*R*)-1-phenylethylamine and chiral (*S*)-1-phenylethanol from the *racemic* amine (Additional file 1: Figure S1) was compared to strains with intact *GPD*. (*R*)-1-phenylethylamine and (*S*)-1-phenylethanol are important synthons for production of various bioactive molecules, and their preparation by whole-cell biocatalysis offers an attractive alternative to chemical synthesis [8]. Herein, the whole-cell biocatalytic activity, NADH regeneration rate, glucose demand, and general physiological response of the engineered *gpd1*Δ*gpd2*Δ yeast also over-expressing a recombinant ω-TA and a KRED are discussed.

## Results

### Construction of whole-cell biocatalysts with over-expressed VAMT and SADH

Native *S. cerevisiae* does not possess any background transaminase activity for (*S*)- or (*R*)-1-phenyethylamine [9], and only a very limited activity for the reduction of acetophenone to (*S*)-1-phenylethanol by endogenous oxidoreductases [8]. A series of active *S. cerevisiae* whole-cell biocatalysts were therefore constructed by over-expressing synthetic codon-optimized genes encoding an (*S*)-selective ω-TA from *C. chinense* (vanillin aminotransferase, VAMT) [24] coupled to a NADH-dependent (*S*)-selective alcohol dehydrogenase (SADH) from *Rhodococcus erythropolis* [25]. VAMT and SADH were chosen based on their substrate specificity and that they have previously been shown to have excellent enantio-selectivity; VAMT for the conversion of (*S*)-1-phenylethylamine to acetophenone [24] and SADH for the conversion of acetophenone to (*S*)-1-phenylethanol [25]. In the case of VAMT, it has also previously been shown to be active in *S. cerevisiae* for the reaction under investigation herein [8, 9].

The codon-optimized synthetic gene coding for SADH was cloned into a yeast integrative plasmid (YIp) either alone or in combination with the codon-optimized synthetic gene encoding VAMT. *SADH* and *VAMT* were cloned downstream of the *PGK1* and *TDH3* promoters, respectively, which are known to be constitutive and highly expressed [26]. The YIp-constructs with only *SADH* (YIpOB7-SADH) or with both *SADH* and *VAMT* (YIpOB7-SADH-VAMT) were transformed in *S. cerevisiae* CEN.PK background strains (TMB4132 and TMB4133) with intact or deleted *GPD1* and *GPD2* genes, resulting in four different active whole-cell biocatalysts (TMB4160–TMB4163) (Table 1). Two additional strains (TMB4140 and TMB4144) with the empty plasmids were also used as negative controls to determine background activity of endogenous enzymes.

**Table 1:** Plasmids and strains used in the study

Strain and plasmids	Relevant genetic features	Description	Reference
Plasmids			
YIplac211	*URA3*		[39]
YIpOB7	*URA3 PGK1p*-*XYL2*-*PGK1t, TDH3p*-*ADH1t*		[31]
YIpOB7-SADH	*URA3 PGK1p*-*SADH*-*PGK1t*		This study
YIpOB7-SADH-VAMT	*URA3 PGK1p*-*SADH*-*PGK1t, TDH3p*-*VAMT*-*ADH1t*		This study
*S. cerevisiae* strains			
TMB4132	CEN.PK2-1C *his3*::YIpJK01 *trp1*::*TRP1* *leu2*::*LEU2 ura3* ^-^		[21]
TMB4133	CEN.PK2-1C *his3*::YIpJK01 *gpd1*::*TRP1* *gpd2*:*LEU2 ura3* ^-^		[21]
TMB4140	TMB4132 *ura3::* YIplac211	Empty plasmid	[21]
TMB4144	TMB4133 *ura3::* YIplac211	Empty plasmid, *gpd1*Δ*gpd2*Δ	[21]
TMB4160	TMB4132 *ura3::*YIpOB7-*SADH*	*SADH*	This study
TMB4161	TMB4133 *ura3::*YIpOB7-*SADH*	*SADH*, *gpd1*Δ*gpd2*Δ	This study
TMB4162	TMB4132 *ura3::*YIpOB7-*SADH*-*VAMT*	*VAMT* and *SADH*	This study
TMB4163	TMB4133 *ura3::*YIpOB7-*SADH*-*VAMT*	*VAMT* and *SADH*, *gpd1*Δ*gpd2*Δ	This study

### Evaluation of the effect of GPD deletion on acetophenone reduction by strains expressing SADH

The effect of deleting the *GPD1,2* genes on the bioreduction step was first evaluated in strains over-expressing only *SADH* (TMB4160 and TMB4161). Cells were grown in aerobic batch mode in shake flasks and harvested in early stationary phase. The cell biomass was subsequently used for whole-cell bioconversion of acetophenone to (*S*)-1-phenylethanol in small-scale glass vials sealed with rubber stoppers to ensure fermentative metabolism. The reaction solution was based on a defined mineral medium with an excess of glucose (50 g/l) to allow for cell growth and thus generation of NADH from anabolic reactions. Sealing of the reaction vessels led to a fast consumption of the initially available oxygen which consequently resulted in an oxygen-limited environment and thus a strong need for NADH oxidation via the glycerol biosynthesis pathway.

**Fig. 1:**
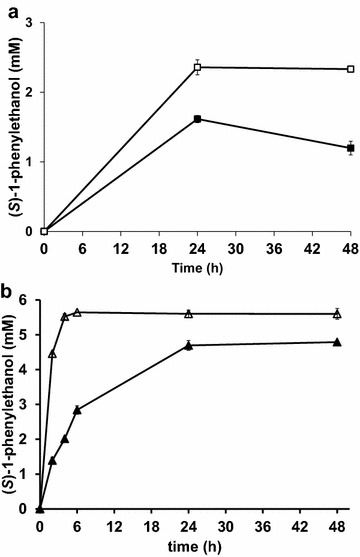
Whole-cell bioconversion of acetophenone to (*S*)-1-phenylethanol. Reactions were catalysed by engineered *S. cerevisiae* strains, over-expressing **a** *SADH* from *R. erythropolis* (TMB4160 and TMB4161); or, **b** *SADH* from *R. erythropolis* in series with *VAMT* from *C. chinense* (TMB4162 and TMB4163). Reaction conditions were as follows: 5 g (dw)/l recombinant *S. cerevisiae*, 5 mM (**a**) or 7.5 mM (**b**) acetophenone, 50 g/l glucose, in defined mineral medium buffered to pH 7. Prior to the reactions, cells were grown in aerobic batch mode with defined medium with glucose as carbon and energy source and harvested by centrifugation in early stationary phase. Symbols: (**a**) *filled squares*, TMB4160 (control, SADH); *open squares*, TMB4161 (*gpd1Δgpd2Δ*, SADH). (**b**) *filled triangles* TMB4162 (control, VAMT and SADH), *open triangles* (*gpd1Δgpd2Δ*, VAMT and SADH). Mean values and standard deviations are calculated from two different whole-cell bioconversions

The conversion of acetophenone to (*S*)-1-phenylethanol was approximately 2-fold higher for the *gpd1*Δ*gpd2*Δ strain over-expressing *SADH* (TMB4161) as compared to the control strain with intact *GPD*-genes (TMB4160) (Fig. 1a). The same trends were observed when comparing the whole-cell bioreduction of acetophenone catalysed by strains over-expressing both *VAMT* and *SADH* TMB4162-63 (3-fold higher activity in the *gpd1*Δ*gpd2*Δ strain) (Fig. 1b, Table 2).

**Table 2:** Specific biocatalyst activities and stereo-selectivities

Strain	Description	Specific whole-cell transamination activity^a^ (µmol (*S*)-1-PEA/h/od)	*ee* (*R*)-phenylethylamine (%)	Specific whole-cell reduction activity^b^ (µmol ACP/h/od)	*ee* (*S*)-1-phenylethanol (%)	Specific SADH activity in cell extract (mU/mg total protein)
TMB4140	Empty plasmid	0	0	n.a.	n.a.	11 ± 3
TMB4144	Empty plasmid, *gpd1*Δ*gpd2*Δ	0	0	n.a.	n.a.	n.a.
TMB4160	*SADH*	0	0	n.a.	>99 %	846 ± 76
TMB4161	*SADH*, *gpd1*Δ*gpd2*Δ	0	0	n.a.	>99 %	380 ± 34
TMB4162	*VAMT* and *SADH*	2.4 ± 0.6	3.6 ± 0.1	45.7 ± 1.3	>99 %	248 ± 37
TMB4163	*VAMT* and *SADH*, *gpd1*Δ*gpd2*Δ	7.5 ± 0.6	21.7 ± 1.0	138.1 ± 2.1	>99 %	511 ± 37

Mean values and standard deviations are calculated from two different whole-cell bioconversions

* ee* at the end of the reaction

^a^ First 4 h of whole-cell bioconversion of *rac*-1-phenylethylamine to acetophenone

^b^ First 4 h of whole-cell bioconversion of acetophenone to (*S*)-1-phenylethanol

**Fig. 2:**
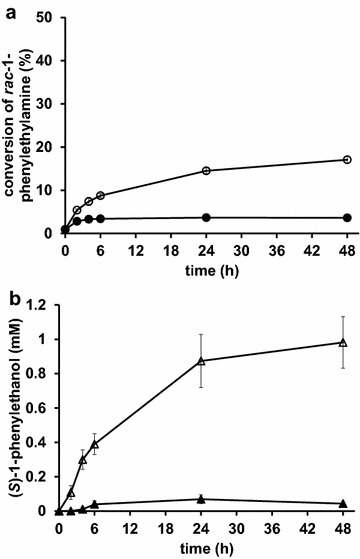
Whole-cell bioconversion of *racemic* 1-phenylethylamine to (*R*)-1-phenylethylamine and (*S*)-1-phenylethanol with cells over-expressing both *SADH* and *VAMT* encoding genes. **a** Conversion (%) of (*S*)-1-phenylethylamine, and **b** concentration (mM) of (*S*)-1-phenylethanol during whole-cell biotransformation of *racemic* 1-phenylethylamine. Reactions were catalysed using recombinant *S. cerevisiae* strains over-expressing *SADH* from *R. erythropolis* and *VAMT* from *C. chinense*, and having native (TMB4162) (*filled symbols*) or deleted (TMB4163) (*open symbols*) glycerol-3-phosphate dehydrogenase (GPD) activity. Reaction conditions were as follows: 5 g (dw)/l recombinant *S. cerevisiae*, 5 mM *racemic* 1-phenylethylamine, 50 g/l glucose, in a defined mineral medium buffered to pH 7. *S. cerevisiae* was grown in aerobic batch mode in stirred tank reactors with defined media with glucose as carbon and energy source and harvested by centrifugation in early stationary phase. Mean values and standard deviations are calculated from two different whole-cell bioconversions

After 6 h of whole-cell bioconversion of acetophenone, the concentration of (*S*)-1-phenylethanol was approximately 5.7 mM and 2.8 mM for the *gpd1*Δ*gpd2*Δ (TMB4163) and control (TMB4162) strains, respectively. The enantiomeric excess (*ee*) of the produced alcohol was >99 % for all *SADH* over-expression strains, indicating limited competing activity from endogenous KREDs with opposite selectivity. This was also demonstrated by an neglectable conversion of acetophenone with the negative control strain (TMB4132) lacking *SADH* (data not shown), which is in accordance with a previously performed study [8]. The substantially higher reductase activity in cell extracts of strains over-expressing *SADH* as compared to the background activity of native reductases (for example YMR226c, [27]) was confirmed by enzyme activity measurements (Table 2). It is noteworthy that although the activity in cell extract was more than 2-fold higher for the control strain (TMB4160) as compared to the *gpd1*Δ*gpd2*Δ strain (TMB4161), the rate of reaction with the metabolically active whole-cell biocatalysts displayed the reversed order of activity, i.e. the conversion with whole-cells was 2-fold higher for the *gpd1*Δ*gpd2*Δ strain. This indicated that the higher catalytic activity in the *gpd1*Δ*gpd2*Δ strain did not result from a higher Sadhp level, but was indeed ascribed to a higher availability of NADH for the reaction in that strain. Additionally, a similar effect was observed when *VAMT* gene was present for TMB4162 (VAMT and SADH) and TMB4163 (VAMT and SADH, *gpd1*Δ*gpd2*Δ), i.e. the double deletion strain displayed 3-fold higher reductase activity than the control strain (Fig. 2). However, in addition to the increased availability of NADH in the *gpd1*Δ*gpd2*Δ strain (TMB4163), a 2-fold higher specific reductase activity in cell extract was also measured (Table 2).

### Whole-cell bioconversion of racemic 1-phenylethylamine to (S)-1-phenylethanol in strains over-expressing SADH and VAMT

The effect of deleting *GPD1,2* on the two-step conversion of (*S*)-1-phenylethylamine enantiomer of the *racemic* amine into acetophenone and then further to (*S*)-1-phenylethanol (Additional file 1: Figure S1) was evaluated. Whole-cell bioconversions of 5 mM *racemic* 1-phenylethylamine were performed under similar conditions as described above, e.g. in defined mineral medium, anoxic conditions, and with an excess (50 g/l) of glucose. Thus, under the applied process conditions, glucose was the sole co-substrate to supply NADH for carbonyl reduction, as well as to supply amine acceptor (for example pyruvate), and the essential prosthetic group PLP for the transamination reaction [9].

The resolution of the racemate to (*R*)-1-PEA was very low for the yeast strain with intact GPD-genes as catalyst (TMB4162) (Fig. 2a), and only a limited conversion of the racemate was obtained (4 % conversion and 4 % *ee* of (*R*)-1-PEA after 24 h). This is in agreement with the 5 % conversion that was observed earlier in a similar background strain only carrying the *VAMT* and where the reaction configurations was with growing cells [8]. The *ee* was however significantly higher than when using control yeast strains as catalyst, since without the expression of *VAMT* there is no conversion at all (Table 2). In summary, this demonstrates that the over-expressed recombinant transaminase was active, although to a relatively low degree. The stereo-selectivity was however high and no conversion of the *R*-enantiomer was detected in any of the experiments made throughout this study, which is in agreement with a previously performed characterization of the enzyme [24]. The low conversion of the amine resulted in formation of acetophenone below the limit of detection and a concentration of (*S*)-1-phenylethanol below 0.1 mM for the control strain (Fig. 2b). This low reaction rate might have been caused by product inhibition of the enzyme, however the acetophenone concentrations obtained were well below the concentration where acetophenone has previously been shown to inhibit VAMT (5 mM) [8]. The low conversion was therefore more likely explained by a low VAMT level and/or limitation in available PLP, since this has been shown previously to influence whole-cell biocatalytic transamination activity [9].

For the *gpd1*Δ*gpd2*Δ strain (TMB4163), the specific whole-cell transamination activity was 3-fold higher as compared to the strain with intact GPD (TMB4162), resulting in a more efficient kinetic resolution (18 % conversion and 22 % *ee* after 48 h), and a final concentration of (*S*)-1-phenylethanol of approximately 1 mM (Fig. 2a–b). The two strains contained the same number of gene copies as determined with qPCR (data not shown). Hence, the difference in activity may therefore not be due to different levels of the recombinant transaminase but rather to a more efficient reduction step.

### Glucose assimilation and regeneration of NADH during reaction progress

**Fig. 3:**
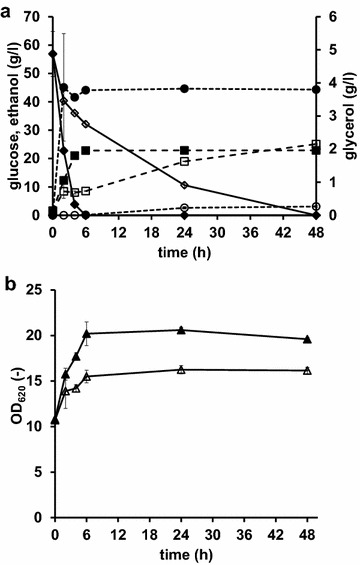
Glucose consumption, metabolite formation, and cell density during conversion of acetophenone to (*S*)-1-phenylethanol with cells over-expressing both *SADH* and *VAMT*. Reactions were catalysed using recombinant *S. cerevisiae* strains over-expressing *SADH* from *R. erythropolis* and *VAMT* from *C. chinense*, and having native (TMB4162) (*filled symbols*) or deleted (TMB4163) (*open symbols*) glycerol-3-phosphate dehydrogenase (GPD) activity. Symbols **a**: glucose (*diamonds*), ethanol (*squares*) and glycerol (*circles*); **b** cell concentration measured as optical density at 620 nm (OD_620_) (*triangles*). Reaction conditions were as follows: 5 g (dw)/l recombinant *S. cerevisiae*, 5 mM *racemic* 1-phenylethylamine, 50 g/l glucose, in a defined mineral medium buffered to pH 7. *S. cerevisiae* was grown in aerobic batch mode in stirred tank reactors with defined media with glucose as carbon and energy source and harvested by centrifugation in early stationary phase. Mean values and standard deviations are calculated from two different whole-cell bioconversions

In accordance with previous studies [21, 28] the rate of sugar consumption differed significantly between the *gpd1*Δ*gpd2*Δ strain and the control strain (Fig. 3a; Table 3). The *gpd1*Δ*gpd2*Δ strain consumed glucose at a 3-fold lower rate than the control strain (29 mM/h vs. 74 mM/h). At the same time the reduction of acetophenone to phenylethanol (Fig. 1) occurred at a higher rate in that strain (Table 2), leading to a higher co-substrate yield for the reduction as compared to the control (Y_P/S_ = 0.05 vs 0.01 mmol chiral alcohol produced/mmol glucose consumed, respectively). The rate of NADH generated from glucose through glycolysis and regenerated via ethanol, glycerol, and 1-phenylethanol production was estimated from their specific production and consumption rates (Table 2). Most of the NADH produced in glycolysis was oxidized by yeast native alcohol dehydrogenase resulting in a significant ethanol production for both strains (Fig. 3a and Table 3). Nevertheless, the ethanol concentration for the *gpd1*Δ*gpd2*Δ strain was significantly lower than for the control, which may have implications for potentially adverse synergistic effect with toxic substrates/products.

**Table 3:** Initial growth rates, rates of substrate consumption, metabolite formation and calculated NADH balance during whole-cell reduction of acetophenone to (*S)*-1-phenylethanol

Strain	Growth rate (h^−1^)	Glucose (mM/h)	Ethanol (mM/h)	Glycerol (mM/h)	Phenylethanol (mM/h)	NADH generation (mM/h)	NADH oxidation (mM/h)
Control, *VAMT* + *SADH* (TMB4162)	0.12 ± 0.02	−74 ± 9.92	105 ± 3.69	9.7 ± 4.52	0.5 ± 0.04	148 ± 19.8	115 ± 5.8
*gpd1*Δ*gpd2*Δ, *VAMT* + *SADH* (TMB4163)	0.07 ± 0.03	−29 ± 9.20	35 ± 14.99	0.03 ± 0.02	1.4 ± 0.25	69 ± 18.4	36.4 ± 15

Rates were calculated for the first 4 h of reaction. NADH balance was calculated from the following assumptions: (i) under anaerobic conditions 2 mol of NADH are theoretically generated per mole glucose consumed. The value is in reality higher since NADH is also generated in anabolic reactions; (ii) 1 mol of NADH is oxidized per mole of ethanol, glycerol or (*S*)-1-phenylethanol being produced. During the initial phase of reaction there was also a low amount of oxygen in the bioreactor which also functioned as electron acceptor in respiration. Mean values and standard deviations are calculated from two different whole-cell bioconversions

Cell growth rate during whole-cell conversion of acetophenone was approximately 2-fold higher for the wild type strain than the double deletion strains (Fig. 3b, Table 3). Furthermore, growth arrested after approximately 6 h of reaction which in the case of the wild type strain coincided with glucose depletion. For the *gpd1*Δ*gpd2*Δ strain there was still >30 g/l of glucose left in the solution and the arrest in growth was instead related to lack of oxygen in the bioreactor. This was in accordance to previous studies where it has been shown that the capacity to proliferate depends on the capacity to oxidize NADH [20, 22]. Nevertheless, growth was not sustained but there was still a significant degree of glucose consumption (Fig. 3a) and thus a significant NADH regeneration throughout the course of reaction (24–48 h).

In fact, the presence of acetophenone or *racemic* 1-phenylethylamine did not influence growth rate or glucose consumption rate during the reactions for any of the strains. With this follows that the substrates were not added at an inhibitory concentration. Furthermore, it also demonstrates that the increased NADH oxidation caused by the SADH-catalysed reduction of acetophenone was insufficient to compensate for the lack of NADH oxidation by GPD1 and GPD2 and thereby insufficient to restore the poor growth of *gpd1*Δ*gpd2*Δ strain under oxygen-limited conditions.

## Discussion

By enhancing the availability of cytosolic NADH through deletion of GPD-catalysed NADH reoxidation, the efficiency of whole-cell reduction of acetophenone, and two-step conversion of *racemic* 1-phenylethylamine to (*R*)-1-phenylethylamine and (*S*)-1-phenylethanol was significantly increased. This demonstrates that the cytosolic NADH generated from anabolic reactions under anaerobic conditions was made available for the SADH-catalysed reduction. Conversion of glucose to ethanol is redox neutral as the 2 mol of NADH generated per mole of glucose in the Embden-Meyerhof-Parnas pathway are oxidized by branching of pyruvate to ethanol, with a theoretical yield of 2 mol ethanol per glucose. Thus, any additional NADH formed during biosynthesis cannot be oxidized by ADH1 that is the main enzyme responsible for regeneration of NAD^+^ by conversion of acetaldehyde to ethanol. Instead, the NADH formed during biosynthesis is oxidized by GPD1- and GPD2-catalysed reduction of DHAP to glycerol-3-phosphate. Consequently, removal of the glycerol pathway decreases the native NADH oxidation capacity, which then leads to a highly reducing intracellular environment which could successfully be diverted in favour of the recombinant NADH—dependent oxidoreductase resulting in significantly higher conversion of acetophenone to (*S*)-1-phenylethanol. Under these conditions it has previously been shown that the limited capacity for NADH oxidation in the *gpd1*Δ*gpd2*Δ strain results in an inability to proliferate under oxygen-limited conditions [20]. However, it has also previously been shown that over-expression of a recombinant xylose reductase and addition of xylose as external electron acceptor could provide *gpd1*Δ*gpd2*Δ yeast with the ability to overcome the hurdle, resulting in improved growth and product formation, i.e. xylitol [21, 22]. An improvement in cell proliferation for the *gpd1*Δ*gpd2*Δ strain during the parallel reaction system under study herein was not observed, thus indicating that the NADH oxidation resulting from the activity of SADH was insufficient to sustain growth. With this follows that there may still be an over-capacity of available NADH in the system, and that an even higher reduction rate might be achievable by further elevation of SADH activity.

The lower formation of biomass under reaction conditions may facilitate down-stream processing, as this previously have been shown to correlate to reduced extraction yields [11]. It is well known that the *gpd1*Δ*gpd2*Δ strain cannot grow under anaerobic conditions and exhibits hampered growth in aerobic shake flask cultivations—traits attributed to deficiency in the strains NADH oxidizing capacity [20]. This has been considered a limiting factor for the exploitation of the strain for biocatalytic purposes. However, recent findings indiciate that the aerobic growth limitatation can be overcome by applying high aereration rates [28]. This allows for an efficient cultivation of the biocatalyst and thus opens further exploitation of the strain.

When only considering the reduction of acetophenone to (*S*)-1-phenylethanol, the co-substrate yield (Y_P/S_, moles product/moles glucose) for the *gpd1*Δ*gpd2*Δ strains over-expressing *SADH* (TMB4161 and TMB4163) was significantly higher than for the control with intact GPD activity (TMB4160 and TMB4162). This was a consequence of reduced glucose consumption combined with increased availability of NADH and higher carbonyl reductase activity. The yield (Y_P/S_ = 0.05 mmol/mmol) was however lower than a previously developed NADPH-accumulating yeast platform system (Y_P/S_ = 0.32 mmol/mmol), in which glucose metabolism was engineered to improve the efficiency of the NADPH-dependent enantio-selective reduction of a bicyclic diketone [14]. Several advantages follow with the decreased glucose flux, e.g. it results in the mentioned lower demand for glucose for the reaction, as well as lower by-product formation. In particular, less ethanol that can be inhibitory to yeast metabolism, and may contribute synergistically to an increased toxicity of reactants or products, is formed.

Acetophenone was converted to (*S*)-1-phenylethanol at a significantly higher rate than *racemic* 1-phenylethylamine was converted to (*S*)-1-phenylethanol when using either the wild type or the double deletion strains. In addition, no acetophenone was detected at any time of the cascade reactions in the *gpd1*Δ*gpd2*Δ strain background, suggesting that it was consumed as quickly as it was formed. Although the increased NADH availability improved the rate of the SADH-catalysed reaction, together the results demonstrate that the rate-limiting step of the two-step conversion of *racemic* 1-phenylethylamine to (*S*)-1-phenylethanol was the initial transamination reaction in the cascade reaction. This observation has been reported previously for a similar system based on the same ω-TA but instead coupled to a NADPH-dependent oxidoreductase from *L. kefir* [8]. It is likely that the reaction cascade can be improved further by elevating the specific whole-cell transamination activity by for example increasing the intracellular VAMT concentration, which have been shown previously to give a positive effect [29]. It has also been shown that addition of the co-factor PLP of the transamination to the reaction solution can improve the reaction [9]. On the other hand, the capacity for PLP-dependent reactions in yeast was previously calculated to be 1.3 mM/gdw/l, which is significantly higher than the maximum rate of transamination reaction achieved in the present study [9].

## Conclusions

In the present study it is demonstrated that by decreasing competition from native NADH-specific oxidative enzymes it was possible to increase the activity of the whole-cell biocatalyst for NADH-dependent transaminase-reductase coupled synthesis and at the same time reduce the requirement for glucose to supply intracellular co-substrates and co-factors. The improvement was shown to be valid both for the NADH-dependent one-step conversion of acetophenone to (*S*)-1-phenylethanol as well as for the two-step conversion of *racemic* 1-phenylethylamine to (*R*)-1-phenylethylamine and (*S*)-1-phenylethanol. The findings made in this work highlight the potential of the *gpd1Δgp2Δ* strain as a potent general platform for asymmetric multistep synthesis by (heterologous) enzymes in which at least one of the reactions is NADH-dependent.

## Methods

### Chemicals

Acetophenone, *racemic* 1-phenylethylamine (1-PEA), and the pure enantiomers (*R*)-1-PEA and (*S*)-1-PEA, all with a purity of ≥98.0 %, were purchased from Merck (Hohenbrunn, Germany). (*R*)-1-phenylethanol and (*S*)-1-phenylethanol were purchased from Sigma-Aldrich (Steinheim, Germany), both with a purity of ≥98.0 %, and all other chemicals were from VWR (Leuven, Belgium).

### Strains and plasmids

*S. cerevisiae* strains and plasmids used in this study are listed in Table 1. *Escherichia coli* NEB 5α (New England BioLabs, Ipswich, MA, USA) was used for subcloning. All strains were stored in 20 % glycerol at −80 °C. Yeast strains were grown on solid YNB media (6.7 g/l yeast nitrogen base without amino acids, 20 g/l glucose and 20 g/l agar) before they were inoculated in the preculture as described below.

### Molecular biology methods

Plasmid DNA was prepared using the GeneJET Plasmid Miniprep Kit (Thermo Scientific, USA). Restriction enzymes and T4 DNA ligase were obtained from the same manufacturer. The QIAquick gel extraction kit (QIagen, Hilden, Germany) was used for DNA extractions from agarose. Nucleotides were purchased from Eurofins (Germany). Genetic constructs were checked by sequencing (Eurofins, Germany). Genomic DNA was extracted with a bead-beater (Biospecs Products, Bartlesville, OK, USA) and phenol/chloroform [30]. PCR was performed with DreamTaq Polymerase (Thermo Scientific, Germany).

### Plasmid construction

Genes encoding *VAMT* [Genbank: AAC78480.1, Uniprot: O82521] [24] and *SADH* [Genbank: AY161280.1, Uniprot: Q6YBW1] [25] were codon-optimized for *S. cerevisiae* and ordered from GenScript (New Jersey, USA). The VAMT containing sequence was digested with *Xba*I, the *SADH* containing fragment was digested with *Bgl*II, and the digested fragments were ligated separately or together with YIpOB7 [31] that had been digested with the same restrictions enzymes resulting in plasmids YIpOB7- SADH and YIpOB7-SADH-VAMT (Table 1). Correct orientation of the inserts and sequences were verified by restriction analysis and sequencing.

### Transformation

*S. cerevisiae* transformations were performed using the lithium acetate method [32], and transformants were selected on defined medium without amino acids or base. *E. coli* transformations were performed according to the Inoue method [33], and selected on Lysogeny Broth (LB) plates [30] with 50 μg/ml ampicillin (IBI Shelton Scientific, Shelton, CT, USA).

### Construction of yeast strains

Plasmids YIpOB7-SADH and YIpOB7-SADH-VAMT were digested with *Apa*I and both transformed separately into TMB4132 and TMB4133 yielding the strains TMB4160 (control, YIpOB7-SADH), TMB4161 (*gpd1Δgpd2Δ,* YIpOB7-SADH), TMB4162 (control, YIpOB7-SADH-VAMT) and TMB4163 (*gpd1Δgpd2Δ,* YIpOB7-SADH-VAMT).

### Quantitative PCR (qPCR)

qPCR was performed with a LightCycler Nano (Roche, Switzerland) to determine relative copy number of the heterologous genes in the constructed strains as described previously [8]. The primers were designed to amplify a region of *VAMT* gene [8]. Genomic DNA was extracted from TMB4160, TMB4161, TMB4162, and TMB4163 using the Yeast DNA Extraction Kit (Thermoscientific, Germany) and the DNA concentrations were measured with Biodrop (Biodrop, Cambridge UK). ExTaq HS polymerase (TaKaRa Bio, Japan) was used in the reaction with DNA-binding dye Eva Green (Biotium, USA).

### Enzymatic assay

Cell extract was prepared with Yeast Protein Extraction Reagent (Thermo Scientific, Pierce, Rockford, USA) according to the instructions provided by the manufacturer. The total protein concentration in cell extracts was determined using the Bradford method [34] with bovine serum albumin (BSA) as standard. SADH activity measurements were performed as described previously [35]. The activity is based on measuring the oxidation of NADH at 340 nm with an Ultrospec 2100 pro spectrophotometer (GE Healthcare Life Sciences, Sweden). The data were collected with the software program SWIFTII (Amersham Biosciences, Sweden). Cell extracts were diluted until the decrease in absorbance was linear for 5 min, at which point the activity could be calculated from the slope. One unit of activity corresponds to 1 µmol NADH consumed per minute at 25 °C. The assay contained sodium phosphate buffer (50 mM, pH 7), acetophenone (10 mM), NADH (0.2 mM), and cell extract (1-30 mg/l total protein).

### Analyses

Growth was followed by measuring the optical density at 620 nm (OD_620_) using a spectrophotometer (Ultrospec 2100 pro, GE Healthcare Life Sciences, Sweden). Glucose, glycerol, ethanol, and acetate were quantified, as described previously [36], with a high pressure liquid chromatography (HPLC) instrument (Waters, Milford, USA) equipped with an Aminex HPX-87H column (Bio-Rad, Richmond, CA, USA) and a RID-10A refractive index detector (Shimadzu, Kyoto, Japan). The mobile phase was 5 mM H_2_SO_4_ and the temperature and flow rate were kept constant at 45 °C and 0.6 ml/min, respectively. (*R*)-1-phenylethylamine, (*S*)-1-phenylethylamine, (*R*)-1-phenylethanol, (*S*)-1-phenylethanol, and acetophenone were quantified as described earlier [8], with a HPLC system (Waters, Milford, USA) equipped with a Daicel Chiralcel OD-H column (4.6 × 25 mm, 5 μm) and a UV/Vis detector 2489. The mobile phase consisted of 85:15 heptane/propan-2-ol with 0.1 % n-butylamine, the flow rate was 1 ml/min and samples were analysed at room temperature.

### Batch cultivation

Cells were pre-cultured overnight in 10 ml defined mineral medium supplemented with 2 % glucose [37], in 50-ml Falcon tubes which were placed in a rotary shaking incubator (Innova 43, Germany) set to 180 rpm and 30 °C. To produce yeast biomass of TMB4140 and TMB4141, cells from the pre-culture were inoculated at an OD_620_ of approximately 0.1 to a baffled shake flask with 50 ml of the same media, and incubated in a shake incubator set to 180 rpm and 30 °C. To produce yeast biomass of TMB4162 and TMB4163, cells from the pre-culture were inoculated at a start OD_620_ of approximately 0.25 for the double deletion strain and OD_620_ of approximately 0.04 for the wild type strain, in a 1-L Multifors bioreactor (Infors, Switzerland) containing 500 ml defined mineral medium (2 % glucose). Cultivations in the Multifors bioreactors were performed at a temperature of 30 °C, a pH of 5.5, a stirring of 800 rpm and with 150 ml/min aeration. Cells were harvested by centrifugation (5000 g for 5 min) in the early stationary phase, and subsequently used for the whole-cell bioconversions.

### Whole-cell bioconversion

Cells were transferred to sealed glass vials containing either 15 or 50 ml defined mineral medium with 50 g/l glucose at an initial OD_620_ of 10. Ergosterol (0.42 g/l) and Tween-80 (0.01 g/l) were added to the vials in order to facilitate anaerobic growth [38] and thus generation of NADH from anabolic reactions. Whole-cell bioconversions were semi-anaerobic and took place in a 30 °C water bath with magnetic stirring. Sampling was made aseptically with a syringe and a needle pierced through a rubber stopper, which also contained an outlet for CO_2_ produced during glucose assimilation. The reactions were started by addition of the substrate, which were either 7.5 mM acetophenone or 5 mM *racemic* 1-phenylethylamine.

## Additional file

10.1186/s12934-016-0430-x Scheme of the studied reactions.
