# Supplementary material for: Increased availability of NADH in metabolically engineered baker’s yeast improves transaminase-oxidoreductase coupled asymmetric whole-cell bioconversion
Source: Microb Cell Fact. 2016 Feb 15;15:37. doi: 10.1186/s12934-016-0430-x (PMC4754910; doi:10.1186/s12934-016-0430-x)
Supplement: Supplementary file 1 — 10.1186/s12934-016-0430-x Scheme of the studied reactions. [file 12934_2016_430_MOESM1_ESM.docx]

**Supplementary material**

**Increased availability of NADH in metabolically engineered baker’s yeast improves transaminase-oxidoreductase coupled asymmetric whole-cell bioconversion**

**Jan Dines Knudsen^1,2^, Cecilia Hägglöf^1^, Nora Weber^1,3^ and Magnus Carlquist^1,*^**

**JDK:** [jan.knudsen@unimib.it](mailto:jan.knudsen@unimib.it), ^1^Division of Applied Microbiology, Department of Chemistry, Faculty of Engineering, Lund University, PO Box 124, SE-221 00 Lund, Sweden. Present address: ^2^The Department of Biotechnology and Biosciences, University of Milano – Bicocca, P.zza della Scienza 4, 20126, Milano (MI), Italy

**CH:** [cecilia.hagglof.350@student.lu.se](mailto:cecilia.hagglof.350@student.lu.se), ^1^Division of Applied Microbiology, Department of Chemistry, Faculty of Engineering, Lund University, PO Box 124, SE-221 00 Lund, Sweden.

**NW:** [noraw@evolva.com](mailto:noraw@evolva.com), ^1^Division of Applied Microbiology, Department of Chemistry, Faculty of Engineering, Lund University, PO Box 124, SE-221 00 Lund, Sweden. Present address: ^3^Evolva, Duggingerstrasse 23, CH-4153 Reinach, Switzerland

**MC:** [magnus.carlquist@tmb.lth.se](mailto:magnus.carlquist@tmb.lth.se), ^1^Division of Applied Microbiology, Department of Chemistry, Faculty of Engineering, Lund University, PO Box 124, SE-221 00 Lund, Sweden.

*Corresponding author email: magnus.carlquist@tmb.lth.se, phone: +46462228329

**
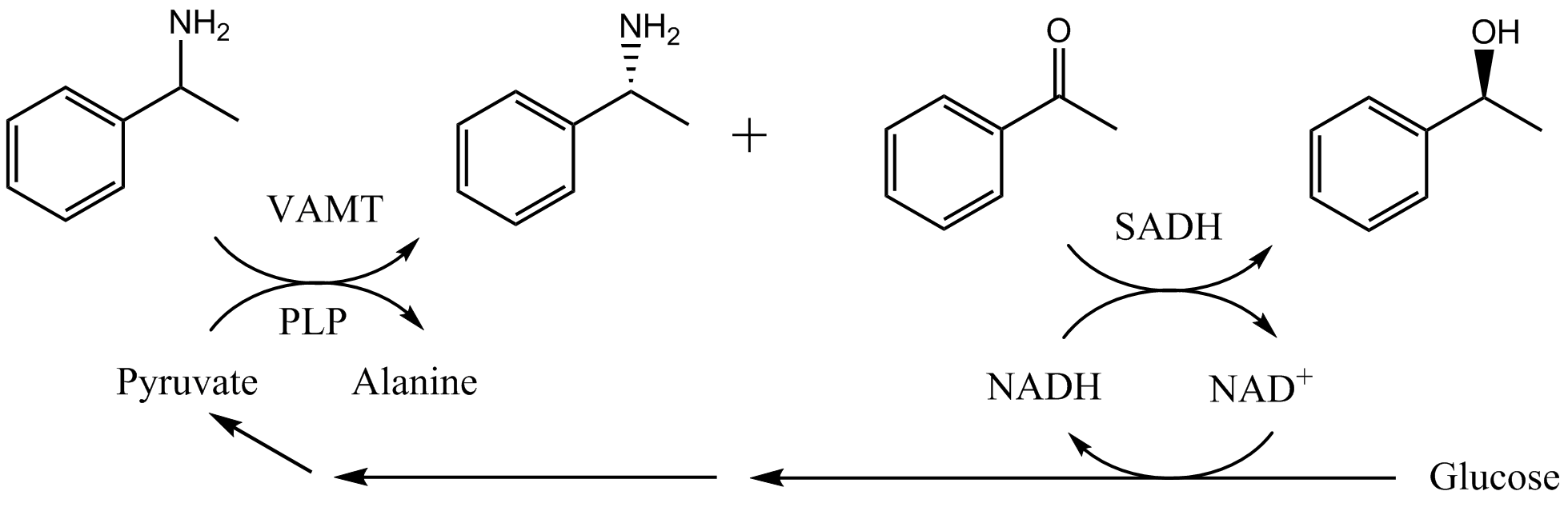
**

Figure S1 Scheme of the studied reactions. The constructed whole-cell biocatalysts were applied for simultaneous (i) kinetic resolution of *racemic* 1-phenylethylamine to (*R*)-1-phenylethylamine and (ii) reduction of the formed acetophenone to (*S*)-1-phenylethanol. Reactions were catalysed using recombinant *S. cerevisiae* strains over-expressing a plant ω-transaminase (*VAMT* from *C. chinense*) and a bacterial ketone reductase (the NADH-dependent oxidoreductase *SADH* from *R. erythropolis*). The whole-cell biocatalyst was produced in an upstream aerobic batch cultivation step, and subsequently used in a semi-anaerobic bioconversion step. The reaction solution was based on a defined mineral medium and consisted of 5-7.5 mM substrate, 5 g (dw)/l engineered yeast, and 50 g/l glucose. The same process configuration was used for one-step conversion of acetophenone or for the two-step conversion of *racemic* 1-phenylethylamine. Pyridoxal-5’-phosphate (PLP), amine acceptor (pyruvate) and NADH were provided by the cell during assimilation of glucose.
